# *Marinobacter atlanticus* electrode biofilms differentially regulate gene expression depending on electrode potential and lifestyle

**DOI:** 10.1016/j.bioflm.2021.100051

**Published:** 2021-06-10

**Authors:** Brian J. Eddie, Anthony P. Malanoski, Elizabeth L. Onderko, Daniel A. Phillips, Sarah M. Glaven

**Affiliations:** a Naval Research Laboratory, 4555 Overlook Ave., SW, Washington, DC, 20375, USA; b Capra Biosciences Inc., 11100 Endeavor Ct., Suite 123, Manassas, VA, 20109, USA; c Oak Ridge Institute for Science and Education / US Army DEVCOM Chemical Biological Center, Biochemistry Branch, Aberdeen Proving Grounds, MD, 21010 USA

**Keywords:** Marinobacter, Bioelectrochemical system, RNAseq, Extracellular electron transfer

## Abstract

*Marinobacter* spp. are opportunitrophs with a broad metabolic range including interactions with metals and electrodes. *Marinobacter atlanticus* strain CP1 was previously isolated from a cathode biofilm microbial community enriched from a sediment microbial fuel cell. Like other *Marinobacter* spp., *M. atlanticus* generates small amounts of electrical current when grown as a biofilm on an electrode, which is enhanced by the addition of redox mediators. However, the molecular mechanism resulting in extracellular electron transfer is unknown. Here, RNA-sequencing was used to determine changes in gene expression in electrode-attached and planktonic cells of *M. atlanticus* when grown at electrode potentials that enable current production (310 and 510 mV vs. SHE) compared to a potential that enables electron uptake (160 mV). Cells grown at current-producing potentials had increased expression of genes for molybdate transport, regardless of planktonic or attached lifestyle. Electrode-attached cells at current-producing potentials showed increased expression of the major export protein for the type VI secretion system. Growth at 160 mV resulted in an increase in expression of genes related to stress response and DNA repair including both RecBCD and the LexA/RecA regulatory network, as well as genes for copper homeostasis. Changes in expression of proteins with PEP C-terminal extracellular export motifs suggests that *M. atlanticus* is remodeling the biofilm matrix in response to electrode potential. These results improve our understanding of the physiological adaptations required for *M. atlanticus* growth on electrodes, and suggest a role for metal acquisition, either as a requirement for metal cofactors of redox proteins or as a possible electron shuttling mechanism.

## Introduction

*Marinobacter* spp. are described as microbial opportunitrophs for their ability to utilize a wide range of carbon substrates in the environment and outcompete other bacteria [1]. As part of their metabolic repertoire, many species have been found to use metals (e.g., iron, arsenic, and manganese) as electron donors [[2], [3], [4]] or acceptors [[5], [6], [7], [8]] during both microaerobic and anaerobic growth. Such observations have led to speculation that *Marinobacter* spp. play a major role in biogeochemical cycling [9]. The isolation of some strains from biocathodes suggested that they may be able to use the electrode as an electron donor in addition to metals [[10], [11], [12], [13], [14], [15]]. However, no mechanism for either metal-oxidation or EET with electrodes has been described that might help explain the role of *Marinobacter* spp. in the environment or promote their use in microbial electrochemical technologies.

*Marinobacter atlanticus* is an aerobic, heterotrophic marine bacterium isolated from an electrode biofilm community called Biocathode MCL (*Marinobacter, Chromatiaceae, Labrenzia*) [11]. Although the role of *M. atlanticus* in the Biocathode MCL community is unknown, in isolation it can produce or consume small amounts of current when grown as an electrode biofilm. Like other *Marinobacter* species, *M. atlanticus* can oxidize or reduce iron when an organic carbon source is also provided, but does not appear to conserve energy for growth and cannot grow autotrophically [6,16]. *M. atlanticus* is a “weak electricigen” [17]; when grown on an electrode at potentials that promote electron uptake, current is at least one order of magnitude lower than the Biocathode MCL community [15]. At potentials that promote current production, current is four orders of magnitude less than what is typically observed for well-studied electricigens like *Geobacter sulfurreducens* [18]*.*

The *M. atlanticus* genome does not contain any large multi-heme redox proteins typically associated with EET and cells do not grow under anoxic conditions when an electrode is the sole electron acceptor [15,16]. Addition of excess trace minerals or riboflavin results in an increase in anodic current [15], suggesting that *M. atlanticus* may utilize trace redox shuttles available in the medium to facilitate EET. Previous metatranscriptomics analysis of the Biocathode MCL community indicated the gene for rubredoxin was highly expressed in *M. atlanticus*, suggesting it may play a role [19]. Mutant strains of *M. atlanticus* where the gene for a rubredoxin and rubredoxin oxidoreductase had been deleted did not display a phenotype for electron transfer [15], indicating that this protein is not required for EET to an electrode. However, compensatory gene expression has not yet been ruled out.

In order to gain insight into the biological processes occurring in *M. atlanticus* electroactive biofilms, we characterized gene expression by RNA-sequencing (RNA-seq) of biofilm and planktonic cells grown in bioelectrochemical reactors at three different electrode potentials (160, 310, and 510 mV vs. the standard hydrogen electrode (SHE)). We also compared gene expression between the wild type (WT) and rubredoxin deletion (Δ*rub*) strains under these conditions to further elucidate the role of rubredoxin, if any, in extracellular electron transfer. We used bioinformatics analyses to identify clusters of genes with similar expression profiles that may share a common physiological role. The electrode potential clearly influenced gene expression for both electrode attached and planktonic lifestyles indicating a connection between current and cell physiology. Results suggest that cells grown at electrode potentials that permit current production have different requirements for metal uptake, biofilm-associated proteins, and protein secretion than those grown at 160 mV. At the same time, genes involved in stress response and DNA repair were more highly expressed in cells grown at 160 mV. These insights into the changes in gene expression resulting from growth on electrodes provide directions for future studies using a more targeted approach such as molecular genetics, and may prove useful in engineering pathways for EET in *Marinobacter* species.

## Results

**Fig. 1:**
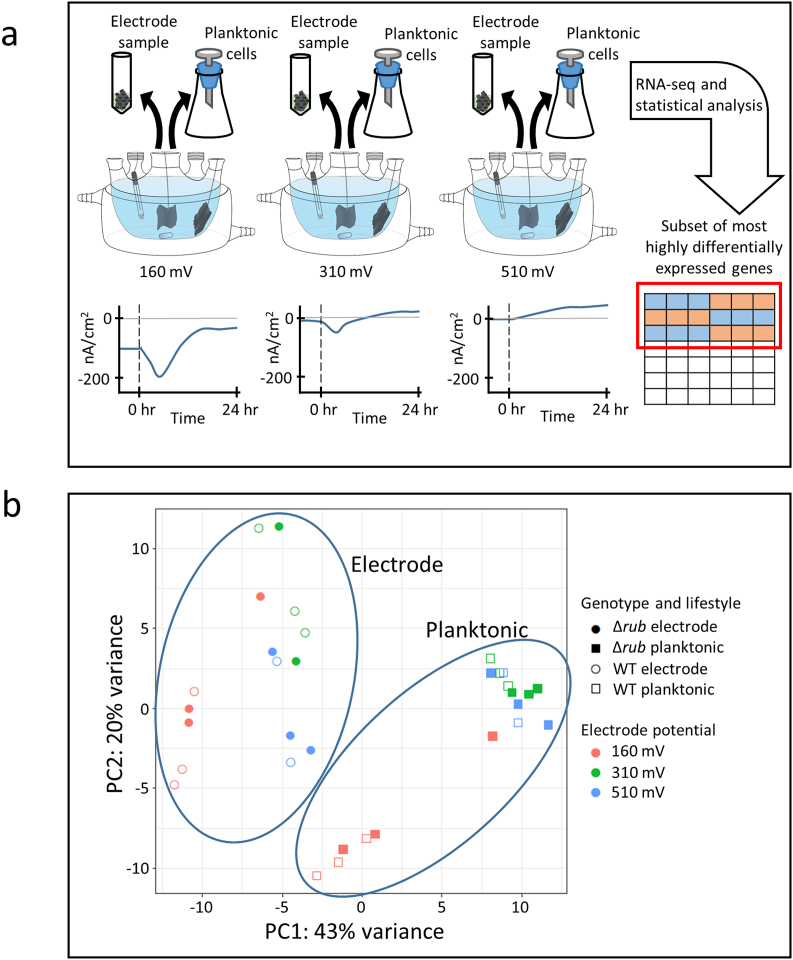
A) Schematic representation of the experiment. Cultures grown at different potentials were sampled by filtration or as the whole electrode. A representative schematic of current is presented at the bottom. The black dashed line indicates when inoculation occurred. B) Principal component analysis of RNA-seq samples with symbols indicating lifestyle: electrode-attached (circles), planktonic cells (squares); genotype: wild-type (open symbols), Δ*rub* strains (closed symbols), and electrode potential: 510 mV (blue), 310 mV (green), and 160 mV (red). (For interpretation of the references to color in this figure legend, the reader is referred to the Web version of this article.)

RNA-seq libraries were prepared from electrode-attached and planktonic cells collected from reactors used during previous electrochemical characterization of *M. atlanticus* [15]. Electrode biofilms were established in the presence of oxygen with succinate as the electron donor and carbon source to promote cellular growth. Maximum current and directionality (current production vs. electron uptake) was dependent on the electrode potential for both the WT and Δ*rub* strains (Fig. 1a), suggesting a physiological response that could manifest as a change in gene expression. At a potential of 160 mV (versus the Standard Hydrogen Electrode (SHE)), biofilms generated negative current until oxygen was depleted [15], at which point current returned to near background, but still negative. This result indicated that cells use the electrode, in addition to succinate, as an electron donor for oxygen reduction. After 24 h of growth, continued cathodic current of −9 nA cm^−2^ may indicate that a small amount of oxygen is leaking into the reactors, facilitating oxygen reduction, or cycling of an unidentified electron mediator. At 310 mV, *M. atlanticus* initially generated negative current, however, once oxygen was depleted, positive current began to increase indicating the electrode was an electron acceptor. At 510 mV, we observed only positive current and maximum current density was higher than at all other potentials. Cells were harvested from reactors after 24–25 h of growth, yielding a total of 36 samples. We selected this time point in order to capture cells at the very beginning of stationary phase where cells are still metabolically active and gene expression is reflective of electron transfer to the electrode more than oxygen reduction. We prepared and sequenced 35 RNA-seq libraries (transcriptomes) yielding over 10^6^ unambiguously aligning reads for each sample (Figure S1). One of the Δ*rub* samples from 310 mV yielded insufficient RNA, and was not sequenced.

*Gene expression is influenced by lifestyle and electrode potential.* Principal component analysis (PCA) of normalized data (DESeq2 methodology for normalization) was first performed to visualize large differences in gene expression between the WT and Δ*rub* strains, lifestyle (biofilm and planktonic), and electrode potential (Fig. 1b). The transcriptomes of both electrode-attached and planktonic cells from one WT reactor at 510 mV were dissimilar from all others, suggesting that this reactor is not directly comparable. We omitted these samples from further analysis to avoid degrading the fit of the data to the statistical model based on recommendations by Gierliński et al. [20]. For the remaining reactors, transcriptomes of electrode-attached cells clearly separated from planktonic cells along principal component 1. Likewise, samples collected from reactors at 160 mV clearly separated from those at 310 mV and 510 mV along principal component 2. Transcriptomes from reactors at 310 mV and 510 mV clustered together, indicating that gene expression was similar at the time of sampling despite initial differences in electrochemical activity between the two potentials. WT and Δ*rub* samples also clustered together, indicating limited variability in gene expression between the two strains and supporting previous electrochemical measurements that showed no difference in current [15].

*Rubredoxin is not involved in extracellular electron transfer.* We compared the transcriptomes of the WT and Δ*rub* strain at each potential to determine if compensatory gene expression could result in current in the absence of rubredoxin (Supplemental Data Set 1). As expected, no transcripts for rubredoxin were detected in the mutant strain. No significant increase in expression (>1.25 log_2_ fold change, p_adj_ < 0.05) of any genes was observed in the Δ*rub* strain relative to the WT in electrode-attached cells at any potential. A single gene predicted to encode a hypothetical protein (ACP86_00815) had significantly lower expression in the mutant strain relative to the WT for both lifestyles across all potentials (Table S1). ACP86_00815 contains a conserved TonB C-terminal domain and appears to be part of a four gene operon with two homologs of the TonB dependent transporter system protein, ExbD (ACP86_00820 and ACP86_00825), and one homolog of ExbB (ACP86_00830). However, *M. atlanticus* did not differentially express genes for ExbD and ExbB.

Twenty-four genes were more highly expressed in the Δ*rub* strain relative to the WT in planktonic cells, though not consistently across all potentials. For example, two genes for alkyl hydroperoxidase (ACP86_08560 and ACP86_08545), the primary scavenger for hydrogen peroxide in *Escherichia coli* [21], were more highly expressed at 310 mV, but not at 160 mV or 510 mV. At 510 mV, an apparent operon (ACP86_06310-ACP86_06320) containing key genes for pyrroloquinoline quinone biosynthesis was more highly expressed in the Δ*rub* strain relative to the WT. Pyrroloquinoline quinone is a redox active bacterial cofactor required for activity of some dehydrogenases [22], and is involved in scavenging reactive oxygen species. Increased expression of alkyl hydroperoxidase and pyrroloquinoline quinone in the Δ*rub* strain may indicate redundant compensation for the absence of rubredoxin in oxidative stress mitigation in planktonic cells [23]. Fifteen genes in planktonic cells of the Δ*rub* strain had decreased expression relative to the WT at a single potential. Five of these were related to nitrogen uptake and assimilation.

Overall, differences in gene expression between the Δ*rub* and WT strains across all experimental conditions were minimal. We did not observe compensatory gene expression related to EET between the two strains in electrode-attached cells, indicating rubredoxin is not involved in EET. Therefore, we combined WT and Δ*rub* transcriptomes to increase statistical power for analyzing differences between potential and lifestyle.

*Functional and regulatory pathways show differential expression by lifestyle and potential*. Functional units associated with lifestyle or electrode potential were identified using gene set analysis [24]. Gene sets are defined by membership to known functional pathways or by genes that are co-expressed under a certain condition, and were generated here using four different approaches: the Kyoto Encyclopedia of Genes and Genomes (KEGG) identifier [25], antiSMASH identifier [26], gene clusters identified from known regulatory networks or systems, and weighted gene co-expression network analysis (WGCNA) [27] (Supplemental Data Set 2). Gene sets based on KEGG pathway identifiers associate genes that share overlapping conserved functional pathways, while antiSMASH associates genes based on secondary metabolite biosynthesis. WGCNA is a statistical approach that creates *de novo* modules of genes from large datasets when they have expression patterns related to each other but may be associated with more than one regulatory network. For example, genes designated to the same WGCNA module may be related because they both consistently experience a change in gene expression at a given electrode potential that is statistically significant across all samples.

**Fig. 2:**
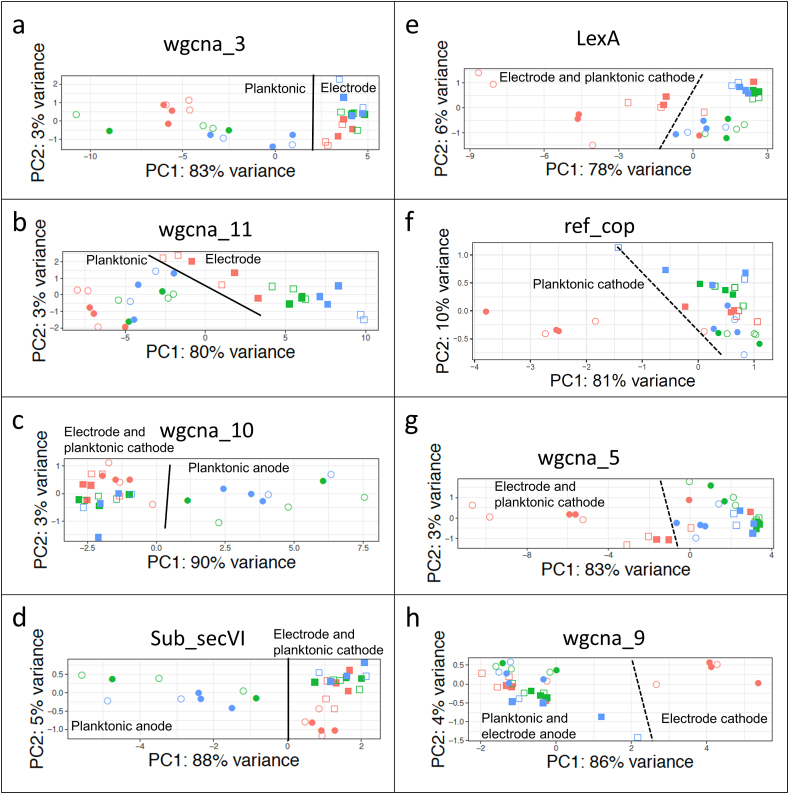
Principal component analysis of gene sets determined to be significantly different using GAGE analysis. A) WGCNA _3, B) WGCNA_11 b C) WGCNA_10, D) KEGG subsystem sub_secVI E) regulon reg_LexA, F) gene cluster ref_cop, G) WGCNA_5, H) WGCNA_9. A solid line notes complete separation of samples from one or more condition, while incomplete separation is noted with a dashed line. Text indicates which samples clustered together. Sample symbols are as in Fig. 1B.

The Generally Applicable Gene-set Enrichment (GAGE) pipeline identified differential gene expression of gene sets [28] (Supplemental Data Set 3). PCA was used to visualize the separation of samples within a gene set where GAGE reported significant differences (Fig. 2). Two gene sets generated by WGCNA, wgcna_11b (221 genes) and wgcna_3 (168 genes), showed lifestyle-dependent expression regardless of potential. Both contain genes predicted to be involved in a wide range of functions including biofilm formation and cell motility. Gene set wgcna_3 included the potential biofilm regulating genes DksA/TraR (ACP86_00795) and a membrane-associated cyclic-diguanylate turnover gene (ACP86_01950), which were more highly expressed in the biofilm. The gene set wgcna_11b had higher expression in planktonic samples and contained genes encoding proteins involved in motility, including the flagellar motor proteins MotAB.

**Fig. 3:**
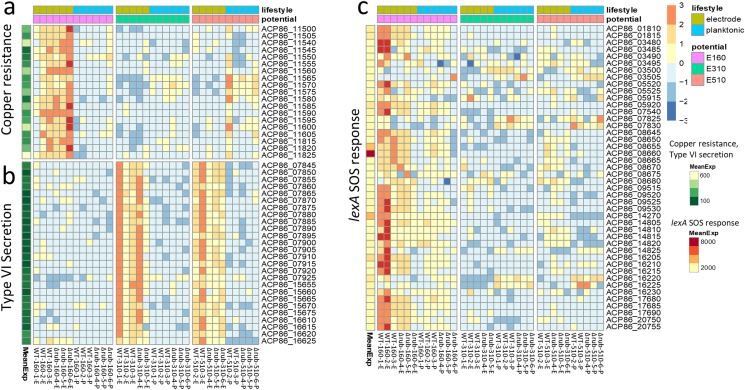
Heatmaps showing relative expression of genes in A) the copper resistance gene set ref_cop, B) type VI secretion system, and C) LexA/SOS response clusters. Base mean expression is given on the left side of each row, with separate scales for Fig. 3a and b and Fig. 3c. Columns are arranged by potential first then by lifestyle as noted by colored boxes at the top. Log_2_ fold change in expression within a gene is given in a blue to red scale. (For interpretation of the references to color in this figure legend, the reader is referred to the Web version of this article.)

Six gene sets showed differential expression in electrode-attached cells based on electrode potential. The type VI secretion system (T6SS) (sub_secVI) and wgcna_10 gene sets were more highly expressed in electrode-attached cells at 310 and 510 mV relative to 160 mV. Twenty-four of the 26 genes in the sub_secVI gene set overlap with the wgcna_10 gene set, indicating a related regulatory network. Four gene sets that may play a role in response to oxidative stress caused by the inability to use the electrode as an electron acceptor — ref_cop, wgcna_9, reg_LexA_SOS, and wgcna_5 — had higher expression in electrode-attached cells at 160 mV relative to 310 and 510 mV. The ref_cop and wgcna_9 gene sets contain genes associated with copper stress and homeostasis and proton and ion transport. Twelve of the 19 genes in the ref_cop gene set overlap with wgcna_9 (28 genes). Gene set reg_LexA_SOS represents the SOS response network regulated by the LexA repressor. LexA modulates expression of the SOS DNA repair mechanism, including expression of the gene for RecA, which is involved in LexA de-repression [29]. Twenty-three of the 44 genes in the reg_lexA_SOS gene set overlap with wgcna_5, including LexA, indicating that they are related. Trends in expression of the individual genes of reg_LexA_SOS, ref_cop, and sub_secVI gene sets confirmed results of the GAGE analysis (Fig. 3).

**Fig. 4:**
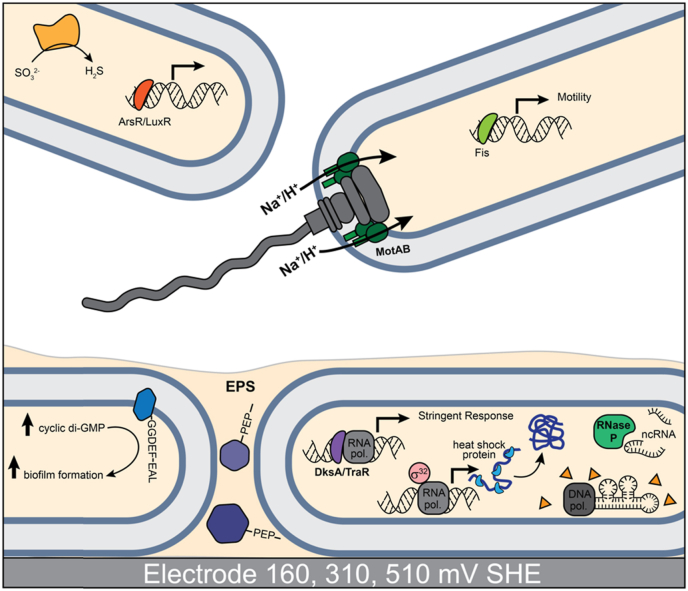
Schematic depiction of biological processes associated with planktonic or electrode attached lifestyles in *Marinobacter atlanticus*. All differentially expressed genes are listed in Table S2. Proteins shown in gray represent other components of multiprotein complexes that are not differentially expressed under these conditions.

*Differential gene expression is evident between electrode-attached and planktonic cells across all potentials*. In order to determine changes in gene expression due to lifestyle only, we compared transcriptomes from electrode-attached cells to those of planktonic cells (Table S2). Sixteen genes were more highly expressed in planktonic cells relative to electrode-attached at all potentials. Two encoded predicted Pom/MotAB proteins (ACP86_10715 and ACP86_10720) associated with the ion motive force transducing mechanism of the flagellar motor complex and determining its ionic specificity (Fig. 4a). In this case, they were not located near the main flagellar operon on the chromosome, which suggests they may play a different role, perhaps in another aspect of ion homeostasis. The gene for a predicted sulfite reductase containing an iron-sulfur cluster and a heme-binding subunit (ACP86_21360) was also more highly expressed in planktonic cells. This protein is usually associated with an NAD(P)H binding subunit that provides the reductant for the six-electron reduction of sulfite or nitrite, but in *M. atlanticus* this subunit is completely absent. In this case, the predicted sulfite reductase was co-transcribed with a putative oxidoreductase gene containing a DUF934 (ACP86_21365), which did not meet the fold change cutoff. An ortholog of this gene has been identified as being required for utilization of sulfate as a sulfur source in *Marinobacter adhaerans* HP15, along with eight other organisms encoding proteins that are predicted to contain this DUF [30].

Three putative transcription factors had increased expression which were annotated as ArsR (ACP86_04765), LuxR (ACP86_06460), and Fis (ACP86_06670) family transcriptional regulators (Fig. 4). The ArsR protein contains a helix-turn-helix motif as is typical of DNA-binding proteins, but also contains an S-adenosylmethionine dependent methyltransferase domain not typically found in the *ars* operon repressor. This architecture is common, with over 6000 representative sequences in the NCBI Protein Family Database, but this family has not been thoroughly investigated. ArsR regulates the arsenical resistance operon in some bacteria [31], however, in *M. atlanticus* it is not located near the genes for the arsenate reductase/transporter operon. The LuxR homolog is an ortholog of the ethanol oxidation regulatory protein ErbR in *Pseudomonas aeruginosa* PA14 (83% amino acid identity) [32,33]. Its potential role in planktonic cells in *M. atlanticus* is unclear. The Fis family regulator may be involved in induction of motility. A set of 17 genes involved in motility and chemotaxis is stimulated by Fis during mid-to late-exponential growth in *E. coli,* and it is known to be autoregulatory, thus tying its own increase in expression to those of its targets [34], suggesting that the increase in expression of ACP86_06670 is a factor in promoting motility in *M. atlanticus* CP1 as well.

Thirty-five genes were significantly more highly expressed in electrode-attached cells relative to planktonic, including many encoding hypothetical proteins. At least two of these have some known association to biofilm regulation and are part of the wgcna_3 gene set. Of these, ACP86_01950 encodes a putative membrane protein that contains the cyclic diguanylate turnover domains GGDEF and EAL (Fig. 4b). Proteins that contain the GGDEF and EAL domains help to regulate transition between sessile and motile phase in bacteria [35]. The other encoded the DksA/TraR family transcriptional regulator (ACP86_00795). Such regulators work by binding RNA-polymerase, where repression or activation is enhanced in the presence of guanosine-5′,3′-tetraphosphate (ppGpp) and/or guanosine-5′,3′-pentaphosphate (pppGpp) [36]. DksA/TraR type transcriptional regulators control expression of a large number of genes as part of the stringent response, including their own expression [37].

In addition, two genes (ACP86_12700 and ACP86_21820) containing PEP CTERM domains putatively associated with biofilm formation were also identified as more highly expressed in biofilms at all potentials. The PEP CTERM domain targets them to the outer membrane and incorporation into the extracellular matrix [38]. The PEP CTERM domain is found much more frequently in biofilm-associated bacteria than in planktonic bacteria, which suggests that the increase in expression of these genes is either a result or cause of the shift to biofilm formation [38].

Six genes have known functions in cellular stress response, including the LexA transcription factor, a prevent-host-death protein (ACP86_02115), RNA polymerase factor sigma-32 (ACP86_07975), and a heat shock protein (ACP86_14050) as well as a protein disaggregation chaperone (ACP86_14050). With the exception of the gene for LexA, all are part of the wgcna_3 gene set. Other genes included those associated with transcription, translation or replication, protein maturation, and metabolism. For example, the RNA processing RNAse P (ACP86_21895) was much more highly expressed (Fig. 4b), possibly indicating that cells have increased non-coding RNA requirements, either for regulation or production of non-coding RNAs like tRNA [39]. Furthermore, four Group II Intron RNA-dependent DNA-polymerases were more highly expressed in the biofilm (ACP86_19365, ACP86_05505, ACP86_13225, ACP86_15860). Similar retroelements have recently been described as an anti-phage bacterial defense system [40]. While regulation of these genes has not previously been associated with biofilm formation, other phage defense systems are controlled by quorum sensing [41].

**Fig. 5:**
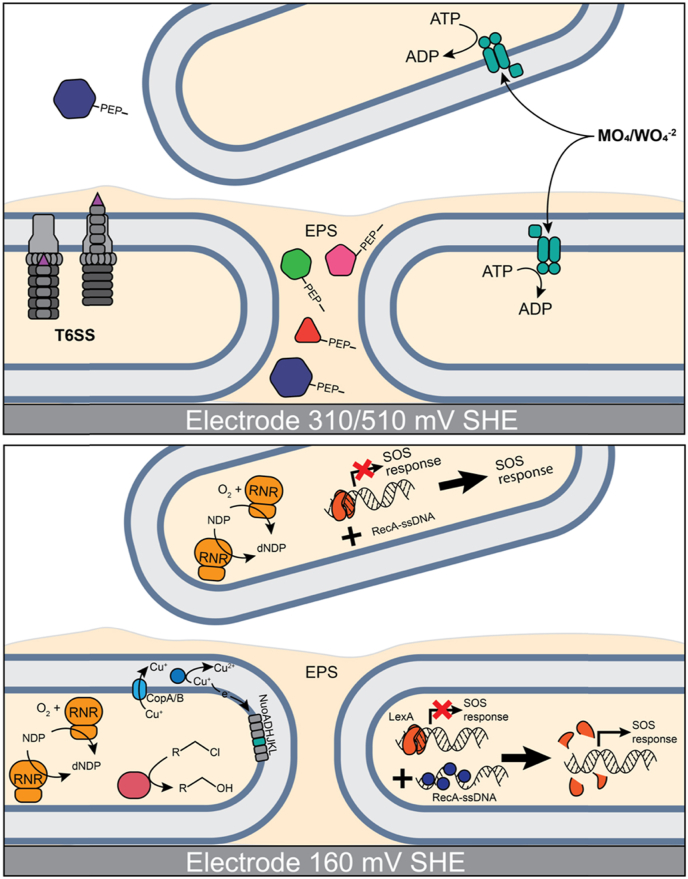
Schematic depiction of cellular activities associated with differences in genes expression between electrode attached and planktonic cells at anodic or cathodic potentials. Predicted cellular functions of genes affected by electrode potential and lifestyle are shown A) between planktonic and electrode attached cells at anodic potentials B) between electrode attached cells and planktonic cells at cathodic potentials. Genes that are differentially expressed between electrode attached cells between the potentials are also depicted. Lists of all differentially expressed genes are in Table S3 and S4. Proteins shown in gray represent other components of multiprotein complexes that do not have differential gene expression under these conditions.

*Oxyanion transport is stimulated during current production.* Previous electrochemical analysis of *M. atlanticus* indicated that planktonic cells do not contribute to current; however, PCA showed that electrode potential influences gene expression in both attached and planktonic cells. Four genes were significantly more highly expressed in both electrode-attached and planktonic cells at 310/510 mV relative to 160 mV (Table S3). Three of these genes (ACP86_11220-ACP86_11230) were co-transcribed and among the top 10% of the most highly expressed genes. They are predicted to be components of an ATP-binding cassette transporter (Fig. 5a). ACP86_11220 encodes a predicted substrate binding protein/sulfate transporter and the amino acid sequence contains six of the seven highly conserved residues critical to selectivity for tungstate over molybdate [42]. The exception is a change in the conserved Threonine-Threonine-Threonine-Serine (TTTS) motif, which is STTS in *M. atlanticus*. Serine/threonine substitutions are among the most common amino acid substitutions, with only minor alterations to the structure and function of a protein [43]. Homology to a characterized oxyanion transporter that is specific for tungstate and molybdate [42], TupABC from *Deltaproteobacteria*, suggest a similar role here. The fourth gene (ACP86_00125) that was more highly expressed at these potentials encodes a hypothetical protein with a PEP C-terminal motif that is associated with sorting proteins to the extracellular side of the outer membrane and was also among the top 10% most highly expressed genes.

*Stress response and DNA repair are stimulated at* 160 mV*.* Nine genes were more highly expressed in both electrode-attached and planktonic cells grown at 160 mV relative to 310/510 mV (Table S3). Most have a role in DNA repair and stress-response. For example, both subunits of a putative ribonucleotide reductase, NrdJ (ACP86_18250 and ACP86_18255) were upregulated. NrdJ is a split nucleotide reductase that is required for anaerobic conversion of ribonucleotides to deoxyribonucleotides for DNA repair [44], and expression is consistent with low oxygen concentrations in the medium at this time point [15]. A gene for the gamma-subunit of exodeoxyribonuclease V (ACP86_09165), also known as RecD, was also more highly expressed. In *E. coli*, RecBCD initiates recombinatorial repair of damaged double-stranded DNA, including that caused by oxidative stress [45]. The genes for the alpha- and beta-subunits, RecBC (ACP86_09155 and ACP86_09160) were not differentially expressed at the cutoff values.

The gene for the LexA transcription factor was more highly expressed in both electrode-attached and planktonic cells grown at 160 mV relative to those grown at 310/510 mV. This indicates that although increased expression of *lexA* is occurring at the electrode for all potentials, the effect is strongest at 160 mV and extends to the planktonic fraction. Three additional genes associated with DNA repair were more highly expressed (ACP86_05920, ACP86_08655, ACP86_08660), including both subunits for an aerobic ribonucleotide reductase (Fig. 5b). Of note, all three belong to the LexA_SOS regulon.

In addition to stress related genes, a haloalkane dehalogenase (ACP86_14360) was more highly expressed at 160 mV relative to 310/510 mV. Haloalkane dehalogenases are enzymes associated with degradation of halogenated alkanes and it is not immediately apparent why expression would be stimulated here. One possibility is that electrochemical side reactions occurring abiotically at the electrode surface result in production of some chlorinated organics that cells metabolize.

*Genes for the T6SS are more highly expressed in electrode-attached cells during current production.* In order to determine gene expression specifically associated with the electrode biofilm during current production, transcriptomes of electrode-attached cells grown at 310/510 mV were cross-compared with planktonic cells grown at 310/510 mV, and then to electrode-attached cells grown at 160 mV (Table S4, Supplemental Dataset 4 and 5). Five genes had increased expression, two of which are part of the T6SS, although not co-located with the main operon. The gene for the major exported protein *hcpA* (ACP86_10175) [46], which forms the penetrating shaft of the T6SS, was much more highly expressed in electrode-attached cells at 310/510 mV relative to 160 mV, and in electrode-attached relative to planktonic cells at 310/510 mV (Fig. 5a). Another gene associated with the T6SS encoding a hypothetical protein (ACP86_16620) was also more highly expressed. No known functional elements are present in the gene sequence, although a homolog of it is associated with the T6SS in *P. aeruginosa* PA14 and is hypothesized to be a toxin similar to colicin [47]. Three genes for hypothetical proteins (ACP86_00120, ACP86_0880, and ACP86_21840) predicted to encode secretion signal peptides and PEP CTERM domains were also upregulated.

*Expression of genes for copper homeostasis and redox stress are stimulated in electrode-attached cells at* 160 mV*.* Thirty-two genes were more highly expressed in electrode-attached cells relative to both planktonic at 160 mV, and to electrode-attached cells at 310/510 mV (Table S5, Supplemental Dataset 4 and 5). All but three belong to either the wgcna_9, ref_cop, wgcna_5 or reg_LexA_SOS gene sets, which is consistent with gene set analysis.

Sixteen of the 28 genes in the wgcna_9 gene set and 10 of the 19 in the ref_cop had differential expression in this comparison, with nine overlapping between the two gene sets. As noted above, genes associated with the wgcna_9 and ref_cop gene sets are involved in copper stress and homeostasis. Overlapping genes with significant differential expression included two genes for the copper resistance protein CopA (ACP86_11500 and 11600) as well as a copper-binding protein (ACP86_11605) (Fig. 5b). In addition to genes involved in copper homeostasis, the ref_cop gene set contains the gene for NuoJ (ACP86_11560), encoding the NiFe-hydrogenase III large subunit, which was also more highly expressed. NuoJ belongs to a cassette of seven colocalized genes (ACP86_11540 - ACP86_11570) which are also in the ref_cop gene set that appear to play a role in ion pumping. Six of the seven are homologous to genes *nuoADHJKL* for the NADH ubiquinone oxidoreductase complex of the respiratory electron transport chain, also known as complex I. The seventh gene (ACP86_11565) is a hypothetical protein that is only found in seven known *Marinobacter* spp. Although only the gene for NuoJ met the 1.25 log-fold cutoff criteria, all seven were significantly more highly expressed at 160 mV on the electrode relative to electrode-attached cells at 310/510 mV or to planktonic cells at 160 mV (Fig. 3).

Seven of the 44 genes in the LexA/SOS regulon (reg_LexA_SOS) and ten of the 95 genes in the wgcna_5 were differentially expressed, with six overlapping. Overlapping genes included the gene for the other major recombinase, RecA (ACP86_16205), and the LexA transcriptional regulator which both regulates expression of *recA* and is modified by it. These results indicate that although the LexA/SOS response was previously noted as more highly expressed in both lifestyles at 160 mV, it was significantly higher in electrode-attached cells. Metabolic genes associated with oxidative stress response are also differentially expressed, including isocitrate lyase (ACP86_0670), which can minimize the buildup of excessive reducing equivalents by redirecting carbon metabolism through the glyoxylate shunt and thus reduce oxidative stress [48].

## Discussion

RNA-seq was used to characterize the molecular underpinnings of EET and adaptations allowing growth as electrode biofilms in *M. atlanticus*. Results presented here clearly show that *M. atlanticus* modulates gene expression based on the electrode potential when grown in bioelectrochemical reactors, demonstrating a physiological link between cells and observed current. Both the electrode potential and lifestyle influenced specific regulatory and functional networks, including the T6SS and the SOS regulon. In addition, new co-expressed clusters identified by WGCNA may indicate previously unrecognized associations between genes. Cellular regulatory networks represent very complex cascades of interactions that may not be directly linked to EET and should be explored further to understand their meaning. These interactions are important considerations for further efforts to engineer electroactive biofilms.

While no clear connection to EET was determined, several interesting observations show that genes for metal uptake and cellular secretion were stimulated at potentials supporting current production. Consistent with electrochemical measurements indicating no significant difference in current or cyclic voltammetry between WT and Δ*rub* strains [15], rubredoxin was not required for generating current. At more positive potentials increased expression of genes for an oxyanion transporter specific for tungstate and molybdate, TupABC, were very highly expressed in both attached and planktonic cells. Increased expression of TupABC suggests trace metals may play at least some role in *M. atlanticus* survivability under these conditions. Addition of excess trace minerals solution, which contains copper sulfate and sodium molybdate but no tungstate, was previously shown to increase current production [15]. Several genes in the *M. atlanticus* genome are annotated as enzymes requiring a molybdate co-factor, including one for a sulfite oxidase (ACP86_21360) that was more highly expressed at 310 and 510 mV, but did not meet the stringent cutoff criteria. Sulfite oxidase is known to catalyze a number of redox reactions inside the periplasm of gram negative bacteria [49]. Copper ions have redox potentials within the ranges observed here, and could potentially play a role as mediators for electron transfer between the cells and the electrodes.

Genes associated with the T6SS system were more highly expressed exclusively in electrode-attached cells at 310 and 510 mV. Gene set analysis predicted the entire T6SS to be significantly more highly expressed; however, most of the individual genes did not meet the applied cutoff limits, with only the major exported protein, HcpA (ACP86_10175), significantly differentially expressed. This is not surprising because these genes typically have relatively low expression in other biofilm-forming bacteria [50]. There are at least two possible explanations for this observation. First, the T6SS was recently implicated in metal scavenging under some conditions, including during oxidative stress in *Burkholderia thailandensis* and iron limitation in *P. aeruginosa* [51,52]. In *B. thailandesis*, Mn^+2^ is obtained using a protein metallophore, TseM (WP_011401419), which is secreted by the T6SS [51]. However, *Marinobacter* spp. do not contain a homolog of this protein. *M. atlanticus* does encode a protein that is homologous to the TseF protein secreted by the T6SS for iron acquisition by outer membrane vesicles in *P. aeruginosa* [52], but this gene (ACP86_01320) is not differentially expressed here. Despite the lack of these specific protein metallophores does not rule out other roles for the T6SS in *M. atlanticus* in metal acquisition. HcpA is found in a conserved arrangement across the *Marinobacter* genus including in *M. manganoxydans, M. salsuginis*, and *M. adhaerans*, along with homologs for a TonB/ExbBD transport system similar to those involved in uptake of iron from TseM in *B. thailandensis* [52]. Here, genes for the TonB/ExbBD transport system have a similar pattern of expression to HcpA and the rest of the T6SS genes, suggesting that they are part of the same process. If T6SS is involved in metal acquisition here, cells may be starved for some metal-cofactor needed during oxygen limitation or increased import of metals such as iron into the periplasmic space could be acting as an electron shuttle with the electrode. Second, secretion of materials to the extracellular matrix is a key component of biofilm formation. While the T6SS is often involved in delivery of toxins in bacterial competition, it can also be activated as part of a general biofilm formation response [53]. The T6SS is associated with growth on hexadecane in *M. hydrocarbonoclasticus*, where it likely contributes to the formation of biofilms at the oil-water interface [54]. The fact that this system is more highly expressed in anode biofilms compared to cathode biofilms may indicate that the anodic potentials are more favorable for cell survivability. This is consistent with previous observations that cathode biofilms were generally less viable based on “live/dead” staining and confocal laser scanning microscopy [15].

The transition from planktonic to attached lifestyles allows a bacterial strain to colonize an electrode and carry out EET [55] and matrix formation is a key component of this transition. *M. atlanticus* encodes eight PEP CTERM domain proteins which are associated with biofilm formation and binding to exopolysaccharides [38,56]. Two of these genes were significantly more highly expressed at all potentials in electrode-attached cells than in planktonic cells, while the other four were only more highly expressed at anodic potentials only. This result is significant because it indicates that the cells in the biofilm are adjusting their surface chemistry in response to substrate potential despite the fact that *M. atlanticus* cannot grow when an insoluble material (i.e., electrode or iron) is the sole electron acceptor [15,16]. Alternatively, changes in the extracellular matrix may provide protection from stressors caused by the electrode potential. The role of PEP CTERM domains has not been fully explored, but they are believed to work in conjunction with exosortase proteins to target transport to the extracellular matrix [57]. The ability to modulate the extracellular matrix has previously been noted as an important consideration for the development of technologies dependent on EET because it is able to act as either a conductive matrix or an insulating layer in addition to its importance for attachment [58].

Cells grown at 160 mV are able to take up electrons from the electrode in the presence of O_2_. Once O_2_ is depleted, current is diminished and cells are electron-acceptor limited. Cell viability is affected as evidenced by previous imaging of the electrode biofilm [15]. Consistent with this, RNA-seq analysis indicated that the cells entered a stress response state and come under metal stress. It is uncertain whether upregulation of both the genes for RecBCD and for RecA/LexA regulon at 160 mV indicates multiple DNA repair pathways activated by potential alone. The increased stress response may be indicative of electron acceptor limitation not experienced by cells at higher potentials that may maintain some basal cellular maintenance by exchanging electrons with the electrode once O_2_ is limiting. We observed a clear link to copper homeostasis and resistance in electrode-attached cells grown at 160 mV. An unusual arrangement of the predicted copper resistance operon was disrupted by the NuoADHJKL operon. NuoADHJKL are commonly found in what is termed a complex I-like arrangement that consists of the ion pumping module and adapter module, with a variable substrate oxidizing module [59]. NuoD is a hydrophilic protein that sits on the cytoplasmic side of the complex, and partially holds the quinone that is the electron acceptor and is part of the adapter that connects the redox enzymes to the physical proton pump. NuoH and NuoA are the other proteins that hold the quinone and are the piston that converts the chemical reaction into physical motion. NuoL is a pump that moves a proton from one side of the membrane to the other when it gets pushed by NuoH and NuoA. NuoL typically has a canonical, long C-terminal extension that acts as a coupling rod to transfer motion to NuoM and NuoN, but this is missing from ACP86_11540 as are the genes *nuoMN*. NuoK can pump a proton as well, suggesting that this complex can translocate two ions per NADH [59]. The substrate that provides the energy for this predicted ion pump is challenging to determine using bioinformatic methods alone, but its proximity and similar expression to the copper resistance genes suggests that it might derive energy from copper redox reactions to pump ions across the cytoplasmic membrane. It is unlikely that these genes form a main component of the electron transport chain, as there is a complete Na^+^ pumping NADH-quinone oxidoreductase complex in the *M. atlanticus* transcriptome with much higher base expression, and little change in expression between conditions. An alternative explanation for this interesting co-localization of genes encoding complex I-like proteins with a cluster of copper resistance genes may be related to a need to replenish local concentrations of redox cofactors when the cell is experiencing a heavy load of copper (or other metal ion) stress. This stress may be more acute at cathodic electrode potentials due to electron acceptor limitation, leading to more off pathway reduction of copper.

## Conclusion

*Marinobacter* spp. are proposed to play a fundamental role in biogeochemical cycling due to their metabolic interactions with hydrocarbons and minerals and their prevalence in metal-rich environments and on electrodes, however, the mechanism for EET remains unknown. Results presented here indicate that genes for protein secretion and metal uptake are stimulated in *M. atlanticus* at electrode potentials where current production is observed, possibly linking EET to use of dissolved minerals as redox mediators. Future exploration of *Marinobacter* electron transfer mechanisms should focus on the role of Mo-dependent enzymes and the T6SS. Changes in expression of genes encoding proteins that are likely targeted to the extracellular matrix or surface of the cells via PEP CTERM domains suggests that *M. atlanticus* remodels its extracellular environment in response to electrode potential. We found several indications that stress response is an important part of adaptation to electrode colonization which will play a role in future efforts to engineer electrode biofilms. Increased stress response at 160 mV may be indicative of electron acceptor limitation not experienced by cells at higher potentials that may support some basal cellular maintenance by exchanging electrons with the electrode once O_2_ is limiting. Further targeted studies are essential for exploring these directions at the molecular level.

## Materials and methods

### Biofilm growth

Samples were obtained from electrodes used for previous electrochemical characterization of *M. atlanticus* CP1 [15]. An experimental workflow and sample key can be found in Figure S1. Briefly, *M. atlanticus* wild type (WT) or the rubredoxin (ACP86_07290) deletion strain (Δ*rub*) were grown aerobically at 30° C in water-jacketed, bioelectrochemical reactors (Pine Research, Durham, NC) in artificial seawater (ASW) medium (n = 3 reactors at each potential). We compared Ag/AgCl reference electrodes (BASi Research Products, West Lafayette, IN) against a new reference electrode with a voltmeter and only used them if they were 10 mV. Stoppers were used no more than two times, because we had previously found this to be a source of variability. Succinate (26 mM) was added as the carbon and primary energy source. Working and counter electrodes were carbon cloth (3 × 6 cm carbon cloth (Zoltek PX30 woven carbon fabric, PW06 weave, Zoltek Corporation) poised at −50, 100, or 300 mV vs. a Ag/AgCl reference electrode. All values in the text are reported versus the standard hydrogen electrode (160, 310, or 510 mV vs. SHE). Following electrochemical characterization, working electrodes were removed and immediately submerged in 5 ml of RNA-later (Thermo-Fisher Scientific, Waltham, MA, USA) in a 50 ml conical centrifuge tube and chilled on ice until all samples were collected. Planktonic cells from 6 to 10 ml of supernatant were collected on a 0.2 μm polycarbonate membrane filter (EMD Millipore, Burlington, MA) using a gentle vacuum. We stopped addition of supernatant once the filter began to clog. We immediately placed the filters into 1 ml of RNA-later and chilled on ice. Once all samples were collected, they were frozen at −80 °C until RNA extraction.

### Biofilm RNA extraction and RNA-seq library preparation

RNA was extracted from both electrode-attached and filtered planktonic biomass using an acid phenol:chloroform:isoamyl alcohol protocol [60]. DNA was degraded by treating 5 μg total RNA with Turbo DNAfree (Thermo-Fisher Scientific), and ribosomal RNA was depleted from 1 μg of DNA-free RNA using a RiboZero Bacteria kit (Illumina, San Diego, CA). Following RiboZero treatment, we purified the samples using the RNA Clean and Concentrate kit (Zymo Research, Irivine, CA) and 5 μl of 20 μl total eluate was used to make RNA-seq libraries. These were constructed with the NEBnext Ultra Directional RNA library prep kit for Illumina (New England Biolabs, Ipswitch, MA) with NEBnext Multiplex Oligo Set 1. Twelve multiplexed libraries were sequenced at a time on a MiSeq (Illumina) using V3 chemistry for 2 × 50 bp paired-end reads. Sequencing and base calling were performed with MiSeq control software version 2.5.1. Raw sequence reads were deposited in the Sequence Read Archive (https://www.ncbi.nlm.nih.gov/sra/) under BioProject PRJNA533641.

### Differential expression analysis of RNA-seq data

The raw MiSeq reads from each sample were trimmed using Trimmomatic 0.33, with the following parameters: HEADCROP:8 LEADING:20 SLIDINGWINDOW:5:20 MINLEN:39 [61]. These trimmed reads were then mapped to the closed and annotated genome of *Marinobacter atlanticus* CP1 (GenBank accession no. CP011929.1) using Bowtie 2 with the “-very-sensitive” parameter [62]. The NCBI GenBank annotation of the genome was used with the program htseq-count to generate counts of reads mapping unambiguously within single features. Statistical significance of differentially expressed genes was determined using the R package DESeq2 version 1.8.1 [63]. Counts were normalized between samples using two methods, DESeq2's internal “estimateSizeFactors” function and the R package cqn 1.14 “glm.offset” function [64].

All samples were processed together and two different linear models were used for the analysis. In the first, condition (electrode potentials 160, 310, and 510), lifestyle (electrode, planktonic) and genotype (WT, Δ*rub*) were considered as factors. In the second, we used only condition and lifestyle, that is, all WT and Δ*rub* samples at a given condition and lifestyle were grouped together. Comparisons were made between the following groups: WT versus Δ*rub* mutant at each electrode potential and lifestyle, electrode versus planktonic at each electrode potential with WT and Δ*rub* grouped together, and the difference between potentials for a given lifestyle with WT and Δ*rub* grouped together. Principal component analysis and distance metrics were determined after the initial DESeq 2 analysis using the regularized log transformation of the normalized read counts which minimizes differences for rows with small counts. Outlier datasets with mean within group (e.g. all WT and Δ*rub* 510 mV electrode samples) log_2_ transformed gene expression correlations of <0.9 were discarded as noted in the results section.

An adjusted p-value (using the Benjamini and Hochberg adjustment [65]) of <0.05, obtained via DESeq2 analysis, was used as the statistical cutoff for differentially expressed genes. The following criteria were used to determine individual genes with significant differential expression associated with lifestyle, potential or both and are described in more detail below: 1) average log_2_ fold change in expression >1.25 for at least one of the three potentials and 2) average log_2_ fold change in expression >1 for the other two potentials and 3) genes with an average expression above the median for the entire transcriptome (normalized read count of 128). The complete list of all comparisons made here is in Supplemental Dataset 6 and corresponding data is in Supplemental Data Sets 1, 4, and 5.

### Pathway and cluster analysis of gene expression

The R package GAGE 2.26 was used to perform generally applicable gene-set enrichment (GAGE) analysis [28] on subsets of genes identified as having functional or regulatory similarity by five different methods, described below. Pathways and modules identified in the *Marinobacter atlanticus* CP1 genome by the Kyoto Encyclopedia of Genes and Genomes (KEGG) were obtained using the R package KEGGREST 1.16 [66]. In the instance of the KEGG secretory system gene set, we manually curated a set of only the genes associated with the T6SS. Three additional genes (ACP86_11615–ACP86_11625) were also included because they appeared to be co-expressed in an operon with ACP86_16610.

The Gene Ontology (GO) terms assigned in the Uniprot database for all predicted proteins from the assembly were downloaded and mapped to the corresponding gene from the output of the htseq-count program to facilitate functional analysis.

Possible regulatory networks were identified primarily by examining the RegPrecise motif database that had identified transcription factor regulons for a group of *Oceanospirillales* and *Alteromonadales* species that are relatively closely related to *M. atlanticus* [67]. Motifs from the database were searched against 350 bases upstream of the start of a translated protein and 50 bases downstream using FIMO from the MEME software suite [68]. Proteins encoded downstream of putative regulatory regions were compared to the proteins in the RegPrecise database regulon to determine the likelihood that the transcription factor exists in this organism and was regulating a similar process.

AntiSMASH bacterial version was used to identify secondary metabolite biosynthetic pathways, using the default settings (detection strictness set to relaxed, KnownClusterBlast, SubClusterBlast, and ActiveSiteFinder on) [26].

Weighted correlation network analysis was performed on the samples using the R package WCGNA 1.63 [27]. The regularized log transforms of the normalized read counts from the DESeq 2 analysis were used as the input for the analysis. A scale-free topology fit determined the best soft-thresholding power to be 6. The dynamic tree cutting method from the R package generated an initial set of modules that were then merged into a final set of modules. The analysis resulted in grouping 1308 genes into 14 different sets. GAGE usually does not provide any significant finding for sets that are very small or very large. For the largest gene set generated, we selected a smaller gene set by filtering for only the genes with the highest potential association to the cluster center, resulting gene sets wgcna_11 and wgcna_11b.

We identified a final gene set after an initial examination of the differential expression. Genes annotated with controlling copper were noted as having a similar expression pattern. Previously published experimental work identified an operon for copper homeostasis in the closely related species *Marinobacter aquaeolei* 617 and was characterized in depth as such for that organism [69]. Prompted by this, we used BLAST search to find genes with high sequence homology in our organism. All the genes had a close match in *M*. *atlanticus,* with most genes still located in one operon. Five genes (ACP86_11500, ACP86_11505, and ACP86_11815- ACP86_11825) were in their own operon in *M. atlanticus*. All the genes were treated as a single gene set for GAGE analysis and it remains a putative regulon. Genes for copper homeostasis flank genes encode a complex I-like structure and appear to be co-regulated, therefore were included in the ref_cop gene set.

## CRediT authorship contribution statement

**Brian J. Eddie:** Conceptualization, Investigation, Methodology, Visualization, Writing – original draft, preparation. **Anthony P. Malanoski:** Methodology, Formal analysis, Visualization, Writing – original draft, preparation. **Elizabeth L. Onderko:** Investigation, Visualization, Writing – review & editing. **Daniel A. Phillips:** Investigation. **Sarah M. Glaven:** Supervision, Writing – original draft, preparation, Funding acquisition.

## Declaration of competing interest

The authors declare the following financial interests/personal relationships which may be considered as potential competing interests: Sarah Glaven reports financial support was provided by Office of the Undersecretary of Defense for Research and Engineering.

## References

[1] Singer E., Webb E.A., Nelson W.C., Heidelberg J.F., Ivanova N., Pati A. (2011). Genomic potential of *Marinobacter aquaeolei*, a biogeochemical “opportunitroph”. Appl Environ Microbiol.

[2] Lysnes K., Thorseth I.H., Steinsbu B.O., Ovreas L., Torsvik T., Pedersen R.B. (2004). Microbial community diversity in seafloor basalt from the Arctic spreading ridges. FEMS Microbiol Ecol.

[3] Balzano S., Statham P.J., Pancost R.D., Lloyd J.R. (2009). Role of microbial populations in the release of reduced iron to the water column from marine aggregates. Aquat Microb Ecol.

[4] Handley K.M., Boothman C., Mills R.A., Pancost R.D., Lloyd J.R. (2010). Functional diversity of bacteria in a ferruginous hydrothermal sediment. ISME J.

[5] Bonis B.M., Gralnick J.A. (2015). *Marinobacter subterrani*, a genetically tractable neutrophilic Fe(II)-oxidizing strain isolated from the Soudan Iron Mine. Front Microbiol.

[6] Wang Z., Eddie B.J., Malanoski A.P., Hervey W.J., Lin B., Strycharz-Glaven S.M. (2015). Complete genome sequence of *Marinobacter* sp. CP1, isolated from a self-regenerating biocathode biofilm. Genome Announc.

[7] Liao S.J., Zhou J.X., Wang H., Chen X., Wang H.F., Wang G.J. (2013). Arsenite oxidation using biogenic manganese oxides produced by a deep-sea manganese-oxidizing bacterium, *Marinobacter* sp. MnI7-9. Geomicrobiol J.

[8] Handley K.M., Hery M., Lloyd J.R. (2009). *Marinobacter santoriniensis* sp. nov., an arsenate-respiring and arsenite-oxidizing bacterium isolated from hydrothermal sediment. Int J Syst Evol Microbiol.

[9] Handley K.M., Lloyd J.R. (2013). Biogeochemical implications of the ubiquitous colonization of marine habitats and redox gradients by *Marinobacter* species. Front Microbiol.

[10] Rowe A.R., Chellamuthu P., Lam B., Okamoto A., Nealson K.H. (2014). Marine sediments microbes capable of electrode oxidation as a surrogate for lithotrophic insoluble substrate metabolism. Front Microbiol.

[11] Wang Z., Leary D.H., Malanoski A.P., Li R.W., Hervey W.J., Eddie B.J. (2015). A previously uncharacterized, nonphotosynthetic member of the *Chromatiaceae* is the primary CO_2_-fixing constituent in a self-regenerating biocathode. Appl Environ Microbiol.

[12] Debuy S., Pecastaings S., Bergel A., Erable B. (2015). Oxygen-reducing biocathodes designed with pure cultures of microbial strains isolated from seawater biofilms. Int Biodeterior Biodegrad.

[13] Erable B., Vandecandelaere I., Faimali M., Delia M.L., Etcheverry L., Vandamme P., Bergel A. (2010). Marine aerobic biofilm as biocathode catalyst. Bioelectrochemistry.

[14] Rousseau R., Santaella C., Bonnafous A., Achouak W., Godon J.J., Delia M.L., Bergel A. (2016). Halotolerant bioanodes: the applied potential modulates the electrochemical characteristics, the biofilm structure and the ratio of the two dominant genera. Bioelectrochemistry.

[15] Onderko E.L., Phillips D.A., Eddie B.J., Yates M.D., Wang Z., Tender L.M. (2019). Electrochemical characterization of *Marinobacter atlanticus* strain CP1 suggests a role for trace minerals in electrogenic activity. Frontiers in Energy Research.

[16] Bird L.J., Wang Z., Malanoski A.P., Onderko E.L., Johnson B.J., Moore M.H. (2018). Development of a genetic system for *Marinobacter atlanticus* CP1 (sp. nov.), a wax ester producing strain isolated from an autotrophic biocathode. Front Microbiol.

[17] Doyle L.E., Marsili E. (2018). Weak electricigens: a new avenue for bioelectrochemical research. Bioresour Technol.

[18] Strycharz S.M., Malanoski A.P., Snider R.M., Yi H., Lovley D.R., Tender L.M. (2011). Application of cyclic voltammetry to investigate enhanced catalytic current generation by biofilm-modified anodes of *Geobacter sulfurreducens* strain DL1 vs. variant strain KN400. Energy Environ Sci.

[19] Eddie B.J., Wang Z., Hervey W.J., Leary D.H., Malanoski A.P., Tender L.M. (2017). Metatranscriptomics supports the mechanism for biocathode electroautotrophy by “C*andidatus* Tenderia electrophaga”. mSystems.

[20] Gierlinski M., Cole C., Schofield P., Schurch N.J., Sherstnev A., Singh V., Wrobel N., Gharbi K., Simpson G., Owen-Hughes T., Blaxter M., Barton G.J. (2015). Statistical models for RNA-seq data derived from a two-condition 48-replicate experiment. Bioinformatics.

[21] Seaver L.C., Imlay J.A. (2001). Alkyl hydroperoxide reductase is the primary scavenger of endogenous hydrogen peroxide in *Escherichia coli*. J Bacteriol.

[22] Shen Y.Q., Bonnot F., Imsand E.M., RoseFigura J.M., Sjolander K., Klinman J.P. (2012). Distribution and properties of the genes encoding the biosynthesis of the bacterial cofactor, pyrroloquinoline quinone. Biochemistry.

[23] Wildschut J.D., Lang R.M., Voordouw J.K., Voordouw G. (2006). Rubredoxin:oxygen oxidoreductase enhances survival of *Desulfovibrio vulgaris* Hildenborough under microaerophilic conditions. J Bacteriol.

[24] Maleki F., Ovens K., Hogan D.J., Kusalik A.J. (2020). Gene set analysis: challenges, opportunities, and future research. Front Genet.

[25] Kanehisa M., Goto S., Sato Y., Furumichi M., Tanabe M. (2012). KEGG for integration and interpretation of large-scale molecular data sets. Nucleic Acids Res.

[26] Blin K., Shaw S., Steinke K., Villebro R., Ziemert N., Lee S.Y., Medema M.H., Weber T. (2019). antiSMASH 5.0: updates to the secondary metabolite genome mining pipeline. Nucleic Acids Res.

[27] Langfelder P., Horvath S. (2008). WGCNA: an R package for weighted correlation network analysis. BMC Bioinf.

[28] Luo W., Friedman M.S., Shedden K., Hankenson K.D., Woolf P.J. (2009). GAGE: generally applicable gene set enrichment for pathway analysis. BMC Bioinf.

[29] Butala M., Zgur-Bertok D., Busby S.J.W. (2009). The bacterial LexA transcriptional repressor. Cell Mol Life Sci.

[30] Price M.N., Wetmore K.M., Waters R.J., Callaghan M., Ray J., Liu H., Kuehl J.V., Melnyk R.A., Lamson J.S., Suh Y. (2018). Mutant phenotypes for thousands of bacterial genes of unknown function. Nature.

[31] Wu J., Rosen B.P. (1991). The ArsR protein is a trans-acting regulatory protein. Mol Microbiol.

[32] Mern D.S., Ha S.-W., Khodaverdi V., Gliese N., Görisch H. (2010). A complex regulatory network controls aerobic ethanol oxidation in *Pseudomonas aeruginosa*: indication of four levels of sensor kinases and response regulators. Microbiology.

[33] Beaudoin T., Zhang L., Hinz A.J., Parr C.J., Mah T.-F. (2012). The biofilm-specific antibiotic resistance gene *ndvB* is important for expression of ethanol oxidation genes in *Pseudomonas aeruginosa* biofilms. J Bacteriol.

[34] Bradley M.D., Beach M.B., de Koning A.P.J., Pratt T.S., Osuna R. (2007). Effects of Fis on *Escherichia coli* gene expression during different growth stages. Microbiology (Read).

[35] Simm R., Morr M., Kader A., Nimtz M., Römling U. (2004). GGDEF and EAL domains inversely regulate cyclic di‐GMP levels and transition from sessility to motility. Mol Microbiol.

[36] Gourse R.L., Chen A.Y., Gopalkrishnan S., Sanchez-Vazquez P., Myers A., Ross W. (2018). Transcriptional responses to ppGpp and DksA. Annu Rev Microbiol.

[37] Chandrangsu P., Lemke J.J., Gourse R.L. (2011). The *dksA* promoter is negatively feedback regulated by DksA and ppGpp. Mol Microbiol.

[38] Haft D.H., Paulsen I.T., Ward N., Selengut J.D. (2006). Exopolysaccharide-associated protein sorting in environmental organisms: the PEP-CTERM/EpsH system. Application of a novel phylogenetic profiling heuristic. BMC Biol.

[39] Kazantsev A.V., Pace N.R. (2006). Bacterial RNase P: a new view of an ancient enzyme. Nat Rev Microbiol.

[40] Millman A., Bernheim A., Stokar-Avihail A., Fedorenko T., Voichek M., Leavitt A., Oppenheimer-Shaanan Y., Sorek R. (2020). Bacterial retrons function in anti-phage defense. Cell.

[41] Høyland-Kroghsbo N.M., Paczkowski J., Mukherjee S., Broniewski J., Westra E., Bondy-Denomy J. (2017). Quorum sensing controls the *Pseudomonas**aeruginosa* CRISPR-Cas adaptive immune system. Proc Natl Acad Sci Unit States Am.

[42] Otrelo-Cardoso A.R., Nair R.R., Correia M.A., Cordeiro R.S.C., Panjkovich A., Svergun D.I. (2017). Highly selective tungstate transporter protein TupA from *Desulfovibrio alaskensis* G20. Sci Rep.

[43] Betts M.J., Russell R.B., Barnes M.R., Gray I.C. (2003). Amino acid properties and consequences of substitutions. Bioinformatics for geneticists.

[44] Torrents E., Sahlin M., Sjöberg B.-M., Andersson K. (2008). The ribonucleotide reductase family—genetics and genomics. Ribonucleotide reductase.

[45] Farr S.B., Kogoma T. (1991). Oxidative stress responses in *Escherichia coli* and *Salmonella typhimurium*. Microbiol Rev.

[46] Filloux A., Hachani A., Bleves S. (2008). The bacterial type VI secretion machine: yet another player for protein transport across membranes. Microbiology (Read).

[47] Hachani A., Allsopp L.P., Oduko Y., Filloux A. (2014). The VgrG proteins are "a la carte" delivery systems for bacterial type VI effectors. J Biol Chem.

[48] Rui B., Shen T., Zhou H., Liu J., Chen J., Pan X. (2010). A systematic investigation of *Escherichia coli* central carbon metabolism in response to superoxide stress. BMC Syst Biol.

[49] Kappler U. (2011). Bacterial sulfite-oxidizing enzymes. Biochim Biophys Acta.

[50] Papenfort K., Förstner K.U., Cong J.-P., Sharma C.M., Bassler B.L. (2015). Differential RNA-seq of *Vibrio cholerae* identifies the VqmR small RNA as a regulator of biofilm formation. Proc Natl Acad Sci Unit States Am.

[51] Si M., Zhao C., Burkinshaw B., Zhang B., Wei D., Wang Y. (2017). Manganese scavenging and oxidative stress response mediated by type VI secretion system in *Burkholderia**thailandensis*. Proc Natl Acad Sci U S A.

[52] Lin J., Zhang W., Cheng J., Yang X., Zhu K., Wang Y. (2017). A *Pseudomonas* T6SS effector recruits PQS-containing outer membrane vesicles for iron acquisition. Nat Commun.

[53] Moscoso J.A., Mikkelsen H., Heeb S., Williams P., Filloux A. (2011). The *Pseudomonas aeruginosa* sensor RetS switches Type III and Type VI secretion via c‐di‐GMP signalling. Environ Microbiol.

[54] Vaysse P.J., Prat L., Mangenot S., Cruveiller S., Goulas P., Grimaud R. (2009). Proteomic analysis of *Marinobacter**hydrocarbonoclasticus* SP17 biofilm formation at the alkane-water interface reveals novel proteins and cellular processes involved in hexadecane assimilation. Res Microbiol.

[55] Rollefson J.B., Stephen C.S., Tien M., Bond D.R. (2011). Identification of an extracellular polysaccharide network essential for cytochrome anchoring and biofilm formation in *Geobacter**sulfurreducens*. J Bacteriol.

[56] Gao N., Xia M., Dai J., Yu D., An W., Li S. (2018). Both widespread PEP-CTERM proteins and exopolysaccharides are required for floc formation of *Zoogloea**resiniphila* and other activated sludge bacteria. Environ Microbiol.

[57] Haft D.H., Payne S.H., Selengut J.D. (2012). Archaeosortases and exosortases are widely distributed systems linking membrane transit with posttranslational modification. J Bacteriol.

[58] Bird L.J., Onderko E.L., Phillips D.A., Mickol R.L., Malanoski A.P., Yates M.D., Eddie B.J., Glaven S.M. (2019). Engineered living conductive biofilms as functional materials. MRS Communications.

[59] Marreiros B.C., Batista A.P., Duarte A.M., Pereira M.M. (2013). A missing link between complex I and group 4 membrane-bound [NiFe] hydrogenases. Biochim Biophys Acta Bioenerg.

[60] Cury J.A., Koo H. (2007). Extraction and purification of total RNA from *Streptococcus mutans* biofilms. Anal Biochem.

[61] Bolger A.M., Lohse M., Usadel B. (2014). Trimmomatic: a flexible trimmer for Illumina sequence data. Bioinformatics.

[62] Langmead B., Salzberg S.L. (2012). Fast gapped-read alignment with Bowtie 2. Nat Methods.

[63] Love M.I., Huber W., Anders S. (2014). Moderated estimation of fold change and dispersion for RNA-Seq data with DESeq2. Genome Biol.

[64] Hansen K.D., Irizarry R.A., Wu Z. (2012). Removing technical variability in RNA-seq data using conditional quantile normalization. Biostatistics.

[65] Benjamini Y., Hochberg Y. (1995). Controlling the false discovery rate: a practical and powerful approach to multiple testing. J Roy Stat Soc B.

[66] Tenenbaum D. (2017). KEGGREST: client‐side REST access to KEGG. R package version 1.16.1.

[67] Novichkov P.S., Kazakov A.E., Ravcheev D.A., Leyn S.A., Kovaleva G.Y., Sutormin R.A., Kazanov M.D., Riehl W., Arkin A.P., Dubchak I., Rodionov D.A. (2013). RegPrecise 3.0--a resource for genome-scale exploration of transcriptional regulation in bacteria. BMC Genom.

[68] Bailey T.L., Johnson J., Grant C.E., Noble W.S. (2015). The MEME suite. Nucleic Acids Res.

[69] Nóbrega F.L. (2009). Heavy-metal resistance in *Marinobacter**aquaeolei* 617 insights into copper resistance.

